# Molecular Analysis of the Interaction of the Snake Venom Rhodocytin with the Platelet Receptor CLEC-2

**DOI:** 10.3390/toxins3080991

**Published:** 2011-08-10

**Authors:** Aleksandra A. Watson, Christopher A. O’Callaghan

**Affiliations:** 1 Department of Biochemistry, University of Cambridge/ 80 Tennis Court Road, Cambridge, CB2 1GA, UK; Email: aaw36@cam.ac.uk; 2 Henry Wellcome Building for Molecular Physiology, University of Oxford/ Roosevelt Drive, Oxford, OX3 7BN, UK

**Keywords:** rhodocytin, CLEC-2, platelets, thrombosis

## Abstract

The Malayan pit viper, *Calloselasma rhodostoma*, produces a potent venom toxin, rhodocytin (aggretin) which causes platelet aggregation. Rhodocytin is a ligand for the receptor CLEC-2 on the surface of platelets. The interaction of these two molecules initiates a signaling pathway which results in platelet activation and aggregation. We have previously solved the crystal structures of CLEC-2 and of rhodocytin, and have proposed models by which tetrameric rhodocytin may interact with either two monomers of CLEC-2, or with one or two copies of dimeric CLEC-2. In the current study we use a range of approaches to analyze the molecular interfaces and dynamics involved in the models of the interaction of rhodocytin with either one or two copies of dimeric CLEC-2, and their implications for clustering of CLEC-2 on the platelet surface.

## 1. Introduction

A range of snake toxins have evolved to influence blood clotting and platelet aggregation [1]. Various C-type lectin-like proteins influence platelet aggregation through interactions with receptors on the surface of platelets; a number of these venom proteins are heterodimeric [2,3]. Platelet activation and aggregation can be triggered by various well defined receptors on the surface of platelets. Under typical physiological conditions, GPIb-V-IX and αIIbβ3 integrin interact with von Willebrand factor when the latter has become attached to extracellular collagen. Collagen itself interacts with the immunoglobulin superfamily receptor GPVI and the integrin α2β1 [4,5]. There is great biomedical interest in the discovery of novel platelet-activating receptors and in this respect the identification of the targets on platelets for snake venom proteins has been an important objective.

The Malayan pit viper *Calloselasma rhodostoma* is a major cause of snakebite morbidity in large parts of Southeast Asia and effects on platelet function are key consequences of envenomation [6]. The pit viper produces a venom protein, rhodocytin (aggretin) which was purified and shown to cause powerful platelet activation and aggregation [7,8]. The molecular cloning and sequence analysis of rhodocytin demonstrated that the two subunits, designated alpha and beta, each have characteristics of the C-type lectin-like family [9]. Rhodocytin has been shown to be a ligand for CLEC-2, a recently identified receptor on the surface of platelets [10]. Binding of rhodocytin to CLEC-2 triggers a potent platelet signaling pathway [10,11]. CLEC-2 contains a single YXXL motif in its cytoplasmic tail. Rhodocytin binding leads to tyrosine phosphorylation in this cytoplasmic tail of CLEC-2, which promotes the binding of spleen tyrosine kinase (Syk), subsequent activation of PLCγ2 and platelet activation and aggregation [10]. CLEC-2 is encoded in a genomic cluster with related C-type lectin-like molecules, some of which have immunological roles, as exemplified by NKG2D [12,13,14,15,16,17]. CLEC-2 was first identified as a receptor on platelets for rhodocytin and may also play a role in lymphatic development [10,18]. We and others have shown that podoplanin is an endogenous ligand for CLEC-2 [16,19]. Podoplanin is expressed on the luminal aspect of lymphatic endothelial cells and in a range of tissues including kidney, heart, lung and many tumours [20]. The biological importance of CLEC-2 and podoplanin is indicated by observations that genetic knockouts of either molecule are lethal during embryonic development [21,22,23,24,25,26,27,28].

The mechanism whereby rhodocytin triggers platelet aggregation is of great interest because a detailed knowledge of CLEC-2-mediated platelet activation could be of value in understanding and preventing platelet aggregation in thrombotic coronary and cerebral vascular disease, which are major causes of disability and death worldwide. Furthermore, there is a strong need to understand the rhodocytin-CLEC-2 interaction in its own right as snake envenomation affects over 2.5 million humans per year, causing more than 100,000 deaths [29]. We have solved the structure of the C-type lectin-like domain of CLEC-2 and have used mutagenesis to characterise the interaction with rhodocytin [30,31]. The binding affinity of CLEC-2 with rhodocytin and podoplanin has been measured using surface plasmon resonance (Biacore technology) [16,30,32]. We found the affinity of the interaction of rhodocytin with monomeric CLEC-2 to be 1.01 ± 0.20 μM, as compared with 24.5 ± 3.7 μM and 4.1 ± 0.2 μM for the interactions of podoplanin with monomeric and dimeric CLEC-2 respectively [16,30,32].

To further explore the association between CLEC-2 and rhodocytin, we have also solved the crystal structure of rhodocytin, and discovered that it assembles as a non-disulfide linked (αβ)_2_ tetramer [33]. Rhodocytin is the first snake venom or other C-type lectin-like protein reported to adopt this configuration. We proposed that the rhodocytin tetramer might induce clustering of CLEC-2 molecules on the platelet surface and that this could play a key role in triggering signaling to platelet activation. We have previously generated three models of the rhodocytin-CLEC-2 interaction, whereby tetrameric rhodocytin may promote clustering of CLEC-2 by interacting with two copies of monomeric CLEC-2, one copy of dimeric CLEC-2, or two copies of dimeric CLEC-2. We have since used a range of cellular, biochemical and biophysical techniques to demonstrate that CLEC-2 exists as a non-disulfide-linked homodimer [32]. Although Syk generally interacts with two YXXL motifs on a single polypeptide chain, there is evidence to indicate that it can interact with two YXXL motifs, each from a different CLEC-2 chain, consistent with CLEC-2 functioning in a dimeric manner in its interaction with Syk [34]. These observations preclude the first of our original models of the rhodocytin-CLEC-2 interaction, wherein two copies of monomeric CLEC-2 bind to tetrameric rhodocytin. In the current study, we analyse and discuss the interactions involved in the two other models of the interaction, where rhodocytin may bind either one or two copies of dimeric CLEC-2, and discuss the implications for clustering of CLEC-2 on the platelet surface.

## 2. Materials and Methods

### 2.1. Protein Interfaces, Surfaces and Assemblies

Algorithms implemented by PISA (Protein interfaces, surfaces and assemblies version 1.2) were used to explore the macromolecular protein interfaces of the model complexes of rhodocytin plus either one, or two copies of model dimeric CLEC-2 [35]. These models were generated as previously reported [33]. These calculations included the structural and chemical properties of macromolecular surfaces and interfaces, the accessible/buried surface area, the free energy of dissociation, and the presence or absence of salt bridges and disulfide bonds. The protein database archive (PDB) was searched for particular interfaces formed by structural homologs, and the PISA database was explored to compare results for multimeric state, symmetry number, space group, accessible/buried surface area, free energy of dissociation, presence/absence of salt bridges and disulfide bonds, homomeric type, and ligands. Structures, interfaces and assemblies were visualised for analysis using Rastop and Jmol (an open-source Java viewer for chemical structures in 3D. http://www.jmol.org/).

### 2.2. Molecular Dynamics

The potential modes of motion of the different models of the rhodocytin-CLEC-2 complex were examined and analysed using the Dynamite package [36]. From the input three dimensional structure, an ensemble of structures was generated, and subsequently analysed to predict which elements move together and the relevant vectors of these motions. The ensemble was generated using Concoord, and the analysis was performed using Gromacs. In essence, Concoord is used to identify all interatomic interactions in the structure which is input [37]. The likely strength of these interactions is analysed and so the potential freedom of the interacting species is modeled to within appropriate bounds. Following this, new variant structures are established that fulfill the limitations of these modeled bounds. These dynamic analyses were represented graphically using Visual Molecular Dynamics (VMD) [38].

## 3. Results and Discussion

### 3.1. The Interaction Surfaces of the Rhodocytin-CLEC-2 Interaction

There are two alpha and two beta subunits of rhodocytin per tetrameric unit. The most basic model of the interaction of rhodocytin with CLEC-2 is that in which one molecule of dimeric CLEC-2 is complexed with one molecule of rhodocytin, the rhodocytin being in the form of the non-disulfide linked (αβ)_2_ tetramer that was identified by crystallography [33]. In this complex, there are two hydrogen bonds and one salt bridge involved in the interaction between dimeric CLEC-2 and one beta subunit of rhodocytin (Table 1). However, in this model, the other beta subunit of the tetramer does not bind to the CLEC-2 dimer (Table 1). In addition to the interactions with the beta subunit of rhodocytin, dimeric CLEC-2 interacts with one alpha subunit of rhodocytin through five hydrogen bonds and three salt bridges (Table 1). In contrast to the beta subunits, where only one interacts with CLEC-2, this second alpha subunit does interact with the CLEC-2 dimer in this model, but does not form any ionic bonds with it (Table 1). It is likely that this interaction with the second alpha subunit is mediated by van der Waals forces.

**Figure 1:**
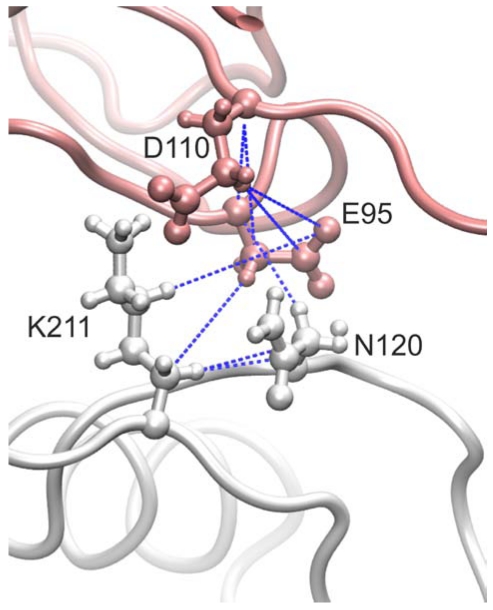
Representation of an interface between CLEC-2 (white) and a beta subunit (pink) of the rhodocytin tetramer. Sidechains of interacting residues (N120 and K211 on CLEC-2, and E95 and D110 on the beta subunit of rhodocytin) are represented as balls and sticks. Predicted hydrogen bonds are represented by broken dotted blue lines.

The more complex model of the rhodocytin-CLEC-2 interaction involves two copies of dimeric CLEC-2 bound to one molecule of the non-disulfide linked (αβ)_2_ tetrameric form of rhodocytin. It is important to note that this model is distinct from that described above, and has been generated independently in a way that has not been influenced by the first model. In this complex, four hydrogen bonds and one salt bridge are involved in the interaction between each molecule of dimeric CLEC-2 and each rhodocytin beta subunit (Table 2). In addition, the interaction between each alpha subunit of rhodocytin and each molecule of dimeric CLEC-2 involves a further two hydrogen bonds and one salt bridge (Table 2). Clearly, this model in which two CLEC-2 dimers associate with tetrameric rhodocytin involves more numerous and favourable ionic interactions and hydrogen bonds (a total of sixteen, as opposed to eleven interactions per tetramer of rhodocytin) in addition to van der Waals contacts and would therefore be a more likely model (Figure 1). Interestingly, residue K150 of CLEC-2, which we identified as being important for its interaction with rhodocytin using mutagenesis and surface plasmon resonance, is also involved in the interface in this model of the complex [30].

There are 246 bound water molecules present in the crystal structure of monomeric CLEC-2 and 45 water molecules present in the crystal structure of rhodocytin. Therefore, upon dimerization of CLEC-2 and complexing of this dimeric CLEC-2 with tetrameric rhodocytin, there will be considerable liberation of the water molecules which solvate the unbound molecules. In our model where one copy of dimeric CLEC-2 binds tetrameric rhodocytin, the alpha subunits and the one beta subunit involved in the complex experience gains in the free energy of solvation of 1.5, 2.5 and 0.2 kcal/mol respectively (total 4.2 kcal/mol). However, in our more complex model in which two CLEC-2 dimers bind to one tetramer of rhodocytin, the solvation energy gains for each CLEC-2 dimer interaction are 0.2 and 0.6 kcal/mol for the alpha subunits, and 2.2 and 0.7 kcal for the two beta subunits (total 7.4 kcal/mol). There is, therefore, a more appreciable gain in the free energy of solvation upon formation of the complex of two CLEC-2 dimers rather than one with tetrameric rhodocytin, which again indicates that this is a more plausible model.

**Table 1:** Contact information and interfacing residues involved in the model interaction of one copy of dimeric CLEC-2 with tetrameric rhodocytin. Key: H = residues making hydrogen bonds, S = residues making a salt bridge, ASA = accessible surface area (Å²), BSA = buried surface area (Å²), ΔiG = solvation energy effect (kcal/mol), |||| = buried area percentage, one bar per 10%.

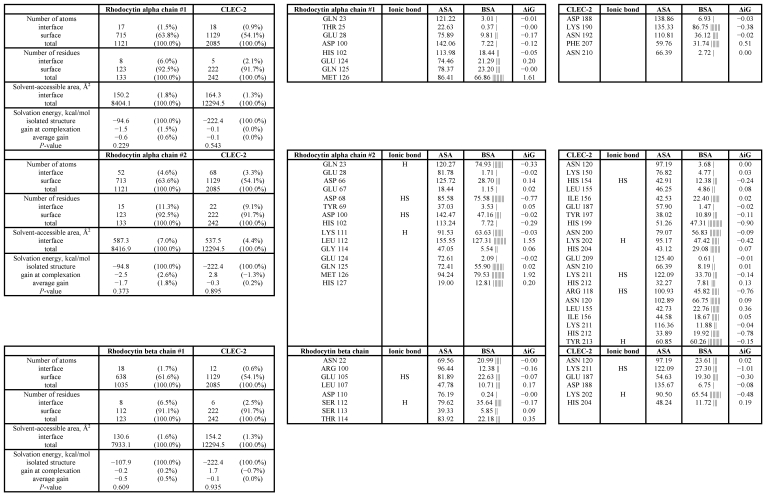

**Table 2:** Contact information and interfacing residues involved in the model interaction of two copies of dimeric CLEC-2 with tetrameric rhodocytin. Key: H = residues making hydrogen bonds, S = residues making a salt bridge, ASA = accessible surface area (Å²), BSA = buried surface area (Å²), ΔiG = solvation energy effect (kcal/mol), |||| = buried area percentage, one bar per 10 %.

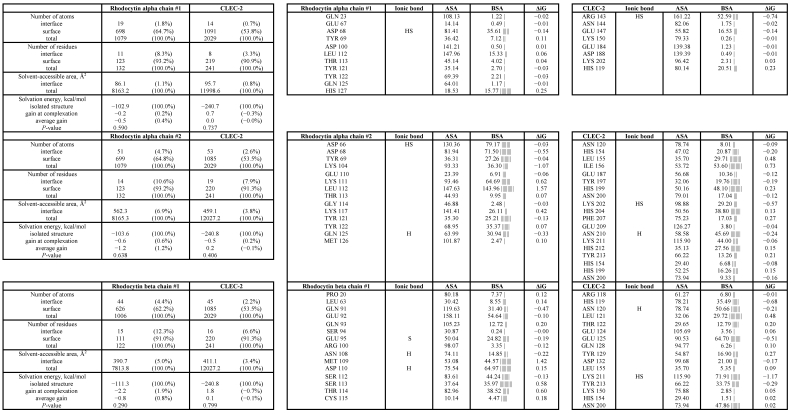

### 3.2. Dynamic Flexibility of the Rhodocytin-CLEC-2 Complexes

**Figure 2:**
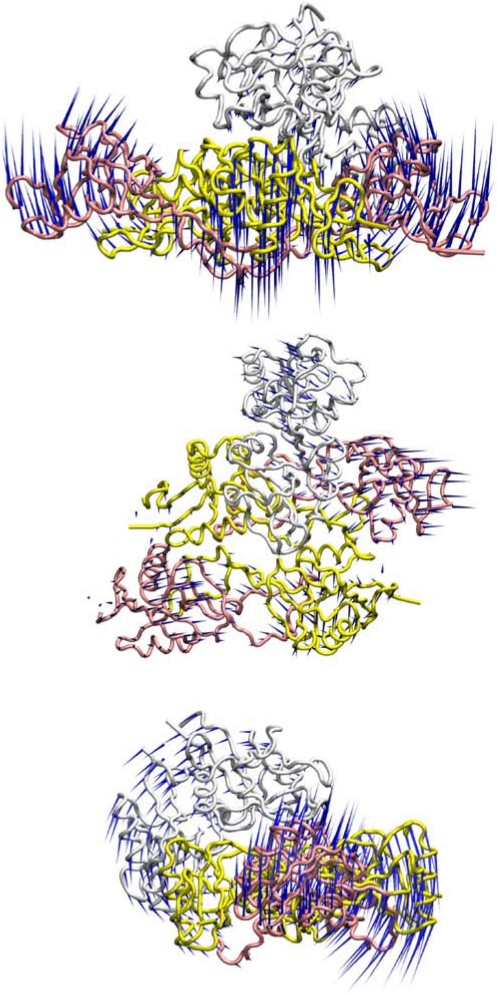
Dynamic analyses of a model of the interaction of dimeric CLEC-2 (white) with tetrameric rhodocytin. The rhodocytin α- and β-chains are coloured yellow and pink, respectively. The porcupine plots represent the principal mode of conformational variability of the Cα atoms calculated from a CONCOORD ensemble using the model of the rhodocytin-CLEC-2 interaction based on the crystal structure of rhodocytin, and a model of the dimeric structure of CLEC-2. Blue cones represent the direction of each motion; the length of the cone is proportional to the amplitude of the motion. The top image represents a 90° counter-clockwise rotation of the central image about the X-axis. The bottom image represents a 90° clockwise rotation of the top image about the Y-axis.

**Figure 3:**
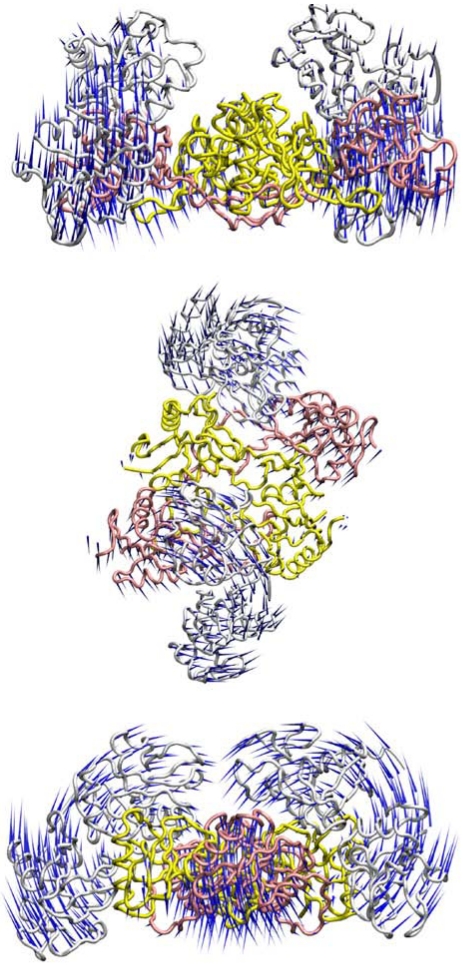
Dynamic analyses of a model of the interaction of two copies of dimeric CLEC-2 (white) with tetrameric rhodocytin. The rhodocytin α- and β-chains are coloured yellow and pink, respectively. The porcupine plots represent the principal mode of conformational variability of the Cα atoms calculated from a CONCOORD ensemble using the model of the rhodocytin-CLEC-2 interaction based on the crystal structure of rhodocytin, and a model of the dimeric structure of CLEC-2. Blue cones represent the direction of each motion; the length of the cone is proportional to the amplitude of the motion. The top image represents a 90° counter-clockwise rotation of the central image about the X-axis. The bottom image represents a 90° clockwise rotation of the top image about the Y-axis.

Molecular dynamics analyses were undertaken to investigate the potential flexibility of the two possible rhodocytin-CLEC-2 interaction models (Figure 2, Figure 3). With only a single dimer of CLEC-2 interacting with tetrameric rhodocytin, the predicted motions are dominated by the alpha and beta subunits of rhodocytin and the motions of CLEC-2 appear to play a relatively minor part in the overall flexibility of the complex (Figure 2). Interestingly, in the model with two copies of dimeric CLEC-2 bound to rhodocytin, a much greater contribution is made by the CLEC-2 dimers, and the beta subunits of rhodocytin, to the global flexibility of the complex (Figure 3). In the molecular dynamics simulations for this model, the CLEC-2 dimers appear to wrap around the grooves presented on rhodocytin, and the beta subunits move in a complementary fashion so as to maximise the exposure and accessibility of the two binding grooves to CLEC-2 (Figure 3). Thus, in this interaction mode with two CLEC-2 molecules, the alpha subunits of rhodocytin make a relatively more minor contribution to the possible dynamic motions of the complex (Figure 3).

## 4. Conclusions

We have previously solved the crystal structures of both rhodocytin and CLEC-2, but there is no crystallographic structure of the rhodocytin-CLEC-2 complex. Using three dimensional structures of both rhodocytin and CLEC-2, we have generated models of the likely modes of interaction of the venom protein and its receptor on platelets and have investigated and analysed the computational models of the interaction which we have generated [30,32,33]. Using a set of analytical algorithms and approaches, we have assessed the properties of the interfacing surfaces and the contribution made to the interaction by specific intermolecular contacts, including salt bridges and hydrogen bonds. In addition to this, we have evaluated the potential flexibility of these model complexes. The model wherein two molecules of CLEC-2 associate with tetrameric rhodocytin provides a more plausible model in terms of the composite effects related to the number of interfacing residues, the nature of their interactions and the predicted solvation energy effects. Further, it is of potential significance that the predicted dynamic motions of this complex are suggestive of a mechanism whereby this interaction might cluster the receptors on the platelet surface, which could have implications for signaling. Overall, the work presented indicates that a plausible mode of binding is that of one non-disulfide linked (αβ)_2_ tetramer of rhodocytin with two dimers of CLEC-2. This analysis will be of value in the development of further studies to characterise the interaction further with a view to developing therapeutic approaches to disrupt the rhodocytin-CLEC-2 interaction on the platelet surface.
